# Exploring the Lived Experiences of Home-Educating Families with Young Children in the UK: The Untold Stories

**DOI:** 10.3390/ejihpe14090171

**Published:** 2024-09-23

**Authors:** Kaili C. Zhang, Lindsay Gibson

**Affiliations:** School of Education, College of Social Sciences, University of Glasgow, Glasgow G3 6NH, UK; lindsay.gibson@glasgow.ac.uk

**Keywords:** home education, homeschooling, UK, portraiture, life course, young learners

## Abstract

Recent trends indicate a significant increase in the number of families opting for home education in the UK, yet research dedicated to this area remains limited. Moreover, there is a notable scarcity of studies focusing on the lived experiences of home-educating families of young children. Amidst this context, a new conceptual framework was developed combining the portraiture approach and the life course theory to study five families with young children in the UK. Semi-structured interviews, observations, and curriculum document analysis were used to gain insights into these families’ experiences. The study revealed diverse motivations driving parents to choose home education, including concerns about the traditional education system, a preference for personalized learning, and personal lifestyle choices and ideologies. The research data presented suggests that participants had diverse and dynamic daily routines shaped by their unique educational philosophies. In general, parents consistently sought beneficial opportunities to foster their children’s social development. Challenges participants faced included curriculum suitability, financial burdens, and managing dual roles within the home. However, participants viewed these challenges as worthwhile because their main aim of educating their children in a purposeful manner was being met. In line with the theme of parental autonomy in education, parents shared their belief in the freedom for families to educate their children outside traditional schools. They supported flexi-schooling, advocated for accessible resources, the inclusion of home-educated children in assessments, and government funding provision.

## 1. Background

### 1.1. Introduction

Elective home education is a term used in the UK to describe the choice made by a parent to provide education outwith the school setting [1]. Any statistical data listed under elective home education does not include children who are too ill to attend school or a child missing education (CME). This also varies from the term flexi-schooling which falls under full-time school education with absence permitted [2].

Recent trends in the UK indicate a significant uptake in the number of families choosing home education as an educational alternative. Indeed, the latest King’s Speech [3] mentioned home education, further indicating this upward trend According to the UK Department for Education [4], the number of homeschooled children has risen significantly over the past two decades, signalling a substantial transformation in educational practices and parental preferences. This trend is corroborated by various studies and reports, including those by the Association of Directors of Children’s Services, as well as home education studies conducted by many researchers in the field [5,6,7].

Shortly after the COVID-19 pandemic, for example, a survey conducted by the Association of Directors of Children’s Services [8] revealed that approximately 115,542 children in England were home-educated during the 2020/2021 academic year. However, government statistics [2] indicate that approximately 92,000 children were registered with local authorities (LA) as being home-educated in October 2023. The DfE acknowledged that this figure might be an underestimation, as it did not account for families unknown to LAs, which is permissible under the current law [9]. At the time of this writing, estimates suggest that up to 180,000 children are currently homeschooled across the four nations in the UK [10,11], making up approximately 1.11% to 1.98% of the 9,073,832 pupils attending schools during the 2023/2024 academic year [4]. Overall, these sources collectively highlight a growing interest among families in alternative educational approaches, leading to an increase in the number of children being educated at home.

As this trend continues to gain momentum, the impact of home education on the broader educational discourse and societal norms is increasingly significant. This phenomenon raises the question: What motivates UK parents of young children to undertake the unique journey of home education and how do they navigate the challenges inherent in this unconventional approach outside mainstream school settings?

### 1.2. UK Context

Similar to many other countries worldwide, home education in the UK remains a contentious issue, with critics expressing concerns about potential social isolation, lack of accountability, and the impact on children’s social and academic development [5,12,13]. Consequently, homeschooling often faces scrutiny and debate, with ongoing efforts to regulate or restrict homeschooling practices [5,14,15].

While conducting a comprehensive review of international studies on homeschooling is beyond the scope of this article, the following sections aim to explore the rising trends and factors influencing parents’ decisions to choose home education. Additionally, by drawing on international perspectives, these discussions seek to enrich our understanding of the unique characteristics and challenges faced by families engaged in home education.

At present, as per the 1996 Education Act in the UK, parents are obligated to ensure that their school-aged child receives “efficient full-time education… either by regular attendance at school or otherwise” [9]. It is also important to note that the specific regulations and guidelines regarding home education may vary slightly across different parts of the UK, as education is a devolved matter, meaning that each country (England, Scotland, Wales, and Northern Ireland) has its own education system and policies. LAs in each area may have their own procedures for monitoring and supporting home-educated children.

The reasons for the surge of homeschooling are multifaceted, encompassing a desire for more personalized learning experiences, dissatisfaction with the mainstream education system (e.g., unhappiness with school values and demands on students), religious convictions, philosophical differences, or inadequate provision of special education needs (SEN) in schools [15,16,17,18]. Additionally, concerns over the social and emotional well-being of children in traditional school settings have also fuelled the rise in home education [13,19,20]. Some parents remove their children from schools due to bullying and school refusal [5], while others are worried about issues such as peer pressure, or negative social influences prevalent in schools. Home education offers parents the option to provide supportive and nurturing learning environments where their children can feel safe, valued, and respected. Furthermore, concerns over the quality and safety of traditional schooling environments, as well as dissatisfaction with standardized testing and curriculum mandates, have led some families to opt for homeschooling [16,21,22].

This growth can also be attributed to various factors, including advancements in technology that have made homeschooling more accessible and convenient, as well as increasing support and resources available to homeschooling families through homeschooling co-ops, online communities, and advocacy organizations [13,23]. Moreover, while conventional views might suggest that students educated in formal school settings would surpass their homeschooled counterparts due to the rigorous training of teachers, research by scholars [23,24,25] in the USA challenges this assumption. Their findings show that homeschooled students tend to outperform their peers on standardized tests, demonstrate notable success in university, and attain higher Grade Point Averages, despite their parents not being subjected to similar educational certifications. Such findings may encourage parents to opt for homeschooling as a viable alternative to traditional schooling. After all, parents have the advantage of understanding the context of their children’s lives in a way that teachers cannot [26].

Nonetheless, despite the growing popularity of home education in the UK, it is crucial not to ignore the distinct challenges confronting home-educating families. The following delineates some of the unique characteristics and obstacles encountered by these families.

### 1.3. Curriculum

Like many other countries where homeschooling is legal, the UK government permits home educators to customize educational experiences to suit their children’s needs, interests, and learning styles. In England, for example, home educators are not obligated to adhere to the national curriculum; however, they are expected to deliver a comprehensive, full-time education for children from the age of five [27].

Many home-educating parents choose a flexible and personalized approach. However, this flexibility can lead to confusion for home educators when selecting suitable curricula. Despite this challenge, home education in the UK is overseen by local councils, which conduct “informal inquiries” to ensure its suitability [4]. They retain the authority to issue school attendance orders if concerns arise, thereby maintaining regulatory oversight [17,18,27].

### 1.4. Funding for Homeschooling Families

Unlike countries such as Canada, New Zealand, and the USA, where government funding for homeschooling is available, there is currently no such provision in the UK. This absence of government funding poses financial challenges for many home-educating families [13]. Consequently, home-educating families in the UK often bear the full financial responsibility for educational resources and many additional support services they may require. This financial responsibility can sometimes limit the educational opportunities available to home-educated students, particularly those from lower-income households. Thus, the absence of government funding for homeschooling in the UK highlights a disparity in support compared to other countries, emphasizing the need to address financial barriers for equitable access to education opportunities and resources.

### 1.5. Socialisation

A look into the international literature shows that inquiries into the socialisation of home-educated children remain prevalent among families, scholars, educators, and the public. As a result, many researchers have addressed this by analysing extracurricular activities, leadership roles, and political participation of individuals educated at home, in state schools, and in private schools from early childhood to young adulthood [12,25,28]. For example, Seiver and Pope’s [12] work demonstrates that homeschooling can provide ample opportunities for socialisation, leadership development, and political engagement, challenging misconceptions and underscoring the multifaceted nature of educational experiences outside of traditional school settings.

In the context of the UK, many home-educating families actively seek out social opportunities through local home education groups, clubs, and activities to ensure that their children have opportunities to interact with peers [13,19]. Some studies have reported that home education allows for meaningful social interactions with family members, peers, and community members, promoting the development of strong social and emotional skills [18,19].

### 1.6. Examinations

Home-educated children in the UK are given the option to take public exams such as the General Certificate of Secondary Education (GCSE), A-levels, and Scotland’s National Qualifications [13,17]. However, accessing these exam centres, which are often located in local schools, presents challenges due to their limited availability for home-educated students [13,17]. This scarcity often necessitates significant travel and incurs additional costs for families opting for national exams. Furthermore, unlike their counterparts attending traditional schools, home-educated students must pay substantial exam fees, adding to the financial burden of home education. The disparity in accessibility to exam centres and the burden of exam fees underscore the unique hurdles faced by home-educated students pursuing formal qualifications. Addressing these challenges is crucial to ensure equitable access to educational opportunities for all students, regardless of their schooling environment.

## 2. This Study

While the literature on home education in the UK has expanded in recent years, a review of the literature reveals a notable gap in in-depth studies focusing on the UK’s home education landscape, particularly concerning the experiences and perceptions of home-educating parents and the impact on young learners [13,29]. To start addressing the mentioned issues, we conducted this qualitative study to gain a comprehensive insight into the experiences of UK home-educating families. Specifically, this study explored the following questions: What are the key motivations for families choosing home education over traditional schooling? How do home-educating families structure their educational practices and daily routines? What challenges, visions, and expectations do families experience in the context of home education? To reflect participants’ changing status and the staged processes they undertake, we developed a new conceptual framework combining the portraiture approach and life course theory.

At this point, it may be useful to briefly explain the key terms used in this study: the terms “home education” or “elective home education” refer to the practice of educating children in a learning environment that is led by parents/guardians rather than a public or private school institution. Parents or guardians take on the primary responsibility for their children’s education during regular school hours, which does not necessarily occur at home. As the term “homeschooling” is widely employed in many other countries, for the purpose of this paper, the term *home education* or *elective home education*, is used interchangeably with “homeschooling”. This is notably different from the term flexi-schooling, which falls under full-time school education with absence permitted [27]. This is a flexible choice that varies depending on what the family decides is best for their child and how willing the school is to accommodate these needs.

## 3. Methods

### 3.1. Theoretical Framework

The study’s theoretical framework needed to reflect participants’ changing status and the staged processes they underwent. It should also account for catalytic interactions between home educators and children, that may at times have driven these changes and processes. To do this, the researchers developed a new conceptual framework combining Lawrence-Lightfoot’s [30] portraiture approach and the life course theory [31,32].

Portraiture involves creating rich, detailed narratives or portraits of individuals or groups studied [30]. Focusing on what is worthy and good, this approach empowers researchers to move beyond mere documentation of facts and statistical analysis to a place of creativity and interpretation, enabling a deeper understanding of the complexities inherent in participants [33]. It is commonly used in fields such as education, psychology, and sociology to investigate participants’ lived experiences. This methodology aligns seamlessly with our goal of understanding the home education experiences of families with young children.

Additionally, the portraiture method offers a platform for expressing divergent views in artful inquiries that traditional positivist research often overlooks [30,34]. Therefore, as the researchers in this qualitative study who intended to act as a human instrument to bring out the voice of the participants, we deemed this stance applicable [35,36]. Another key feature of portraiture is the emphasis on the researcher’s reflexivity and self-awareness. Unlike traditional research methods that emphasize detachment, portraiture emphasizes the relationship between the researcher and the participants, acknowledging the influence of the researcher’s perspective on the study [37].

The life course theory, an interdisciplinary framework exploring factors shaping individuals over time, encompasses stages of life and considers personal experiences, social contexts, and life-altering choices [31,32]. Key elements include transitions, turning points, and career pathways. In our study, the term “transition” signifies significant life changes, emphasizing timing, sequencing, and cumulative effects within interconnected lives. Examples range from marriage to career shifts. *Turning points*, representing pivotal changes in life, offer insights into protective factors and life processes. The life course theory’s framework for *career pathways* recognizes non-linear, decision-laden trajectories influenced by life experiences, contexts, and factors that shape individuals’ career development and choices.

### 3.2. Methodology

In this study, our emphasis was on home-educating parents of young children, aligning with the broader context of early childhood care and education. This study is based on five portraits featuring home-educating families of young children in the UK, with three families based in England and two in Scotland. Home-educating families were eligible for participation in the study if they had been homeschooling for more than three years and had at least one child aged nine or under. Ethical approval to conduct this study was obtained, and all participants provided informed consent to partake in the study and were assured of their anonymity and right to withdraw their data up to the point of analysis. To protect the privacy of the participants, all names used in the paper are pseudonyms.

Acknowledging the importance of involvement and subjectivity in the interpretive process, this study considers each home education setting as an “interpretive community” ([38], p. 7). In our roles as researchers, the authors recognized that each home-educating setting possesses its own unique characteristics and ideologies, distinct from their own. This understanding was crucial in acknowledging and embracing the diversity inherent in the various home education settings encountered during the research process.

Taking on a participant observer role, the first author completed field notes with each family once a week for six weeks. Specifically, semi-structured interviews were conducted with each home-educating family every week, and at least three class or activity sessions (e.g., outdoor play sessions with other children, parent-led in-home experiments) were observed during the six-week period. All conversations were audio-recorded. The settings for each interview and observation were selected by the participants to ensure that both the home educators and their children felt at ease and comfortable sharing their experiences.

The qualitative data analysis, drawn from interviews, observations, and document analysis, revealed key themes representing each participant’s experiences. These themes highlight the complex interplay of construction and interpretation within their journeys [39]. The primary goal was to capture the essence of the “uniqueness” inherent in each home-educating family’s experiences. To convey immediate impressions of the home education settings and experiences, passages introducing each portrait are presented in the present tense, offering current insights into the dynamic nature of the observed settings. Selected excerpts from participants’ answers are included to highlight their voices.

### 3.3. Participants

In qualitative research, sampling prioritizes capturing participants’ experiences over constructing culturally or socially representative samples, typically employing small sample sizes [35]. Therefore, the participants in this study were purposefully selected to provide a diverse and representative snapshot of the home education landscape in the UK.

The gatekeeper-referral and snowballing methods were used to identify potential participants. Initially, referrals from two gatekeepers associated with home education agencies where families of young children can register were utilized [35]. The gatekeepers provided referrals to potential participants, resulting in the recruitment of nine participants. Concurrently, snowball sampling commenced with these nine participants reaching out to individuals in their networks to gauge interest. This process led to three families being referred by two additional participants, resulting in a total of 12 potential participants initially identified. Three families were randomly selected for the pilot study, leaving nine for the main study. However, due to logistical constraints, five families participated, which is an acceptable sample for a portraiture study of this scale to achieve qualitative thematic saturation as we sought to document and illuminate the complexity and detail of the unique experience of each family. The participants’ background characteristics are shown in Table 1.

### 3.4. Research Process

**Figure 1:**
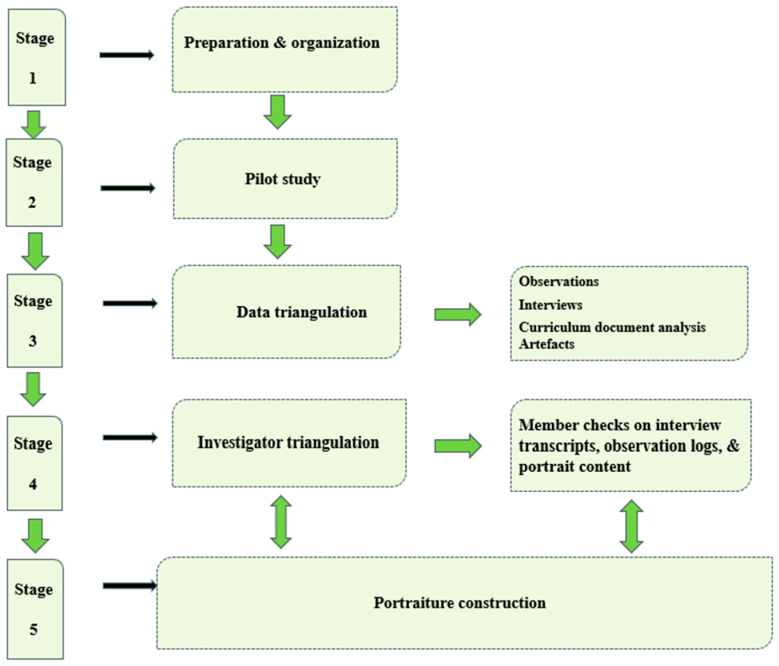
Research process.

The research process, which involved five stages, is illustrated in Figure 1.

**Pilot Study.** A pilot study was conducted with three home-educating parents to ensure the effectiveness and appropriateness of the research design. This involved testing interview protocols (Appendix A), refining questions for clarity, and assessing the feasibility of observational methods. Feedback from participants informed adjustments to the interview structure, ensuring sensitivity to the subtleties of home education experiences.

**Data Triangulation.** Data triangulation, using various sources including interviews, observations, artefacts, and document analysis, aimed to enhance trustworthiness by capturing the complexity of participants’ experiences. It also served as a methodological compass, guiding the exploration of home education experiences, and fortifying the trustworthiness of findings. In-depth interviews provided rich, personal narratives on motivations, challenges, and dynamic evolution of home education journeys, allowing participants to articulate experiences authentically. In addition to interviews, observational methods captured the daily nuances of home education environments and learning activities outside the homes, thereby providing context-rich insights into pedagogies and children’s experiences. Examining the curriculum documents home educators used unearthed contextual signifiers and added depth to the interpretation of educational strategies. Artefacts from home education environments served as tangible representations of educational endeavours, providing a visual dimension to participants’ pedagogical approaches.

In our data triangulation approach, we aimed to provide a multifaceted understanding of home education. Consistency and convergence in narratives across methods added robustness, with discrepancies thoroughly explored among the research team members. Transparency was maintained through detailed documentation and ongoing reflections.

### 3.5. Data Analysis Process: Portrait Construction

**Figure 2:**
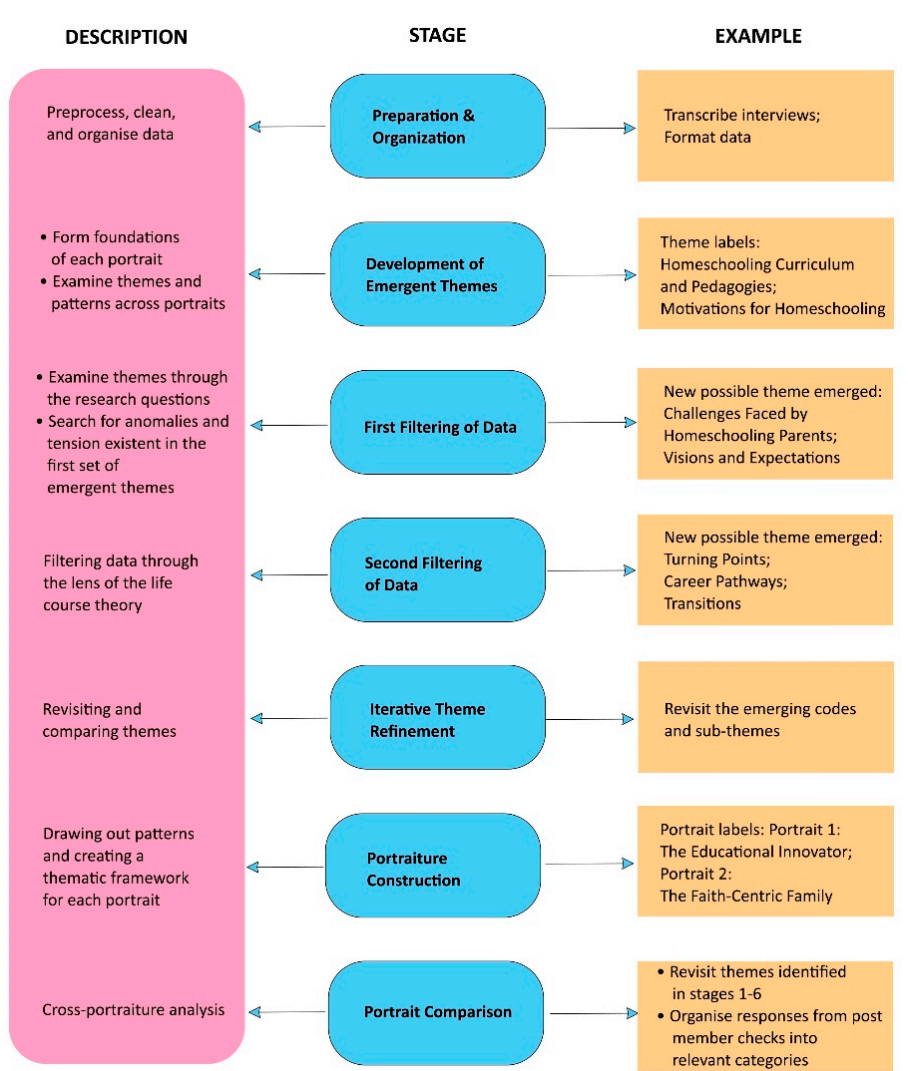
Methods for analysing data.

In each stage of the data collection, themes and patterns were explored as they emerged. The data were then analysed for cross-cutting themes and patterns across the portraits based on the research questions and filtered through the life course theoretical framework. The iterative theme refinement stage involved revisiting and comparing themes by examining emerging codes and sub-themes. Member checks on interview transcripts, observation logs, and portrait content were carried out among researchers on the team. In the portraiture construction stage, patterns were drawn out, and a thematic framework was created for each portrait. Portrait comparison included cross-portraiture analysis, revisiting themes from stages 1–6, and organizing post-member check responses into relevant categories. The data analysis process is illustrated in Figure 2.

Analysis of the interview transcripts revealed a spectrum of motivations for home-educating. In the sections below, detailed narratives of five home education portraits in the UK are presented. Each portrait highlights the unique motivations, experiences, and educational approaches of different families who have chosen to homeschool. These narratives provide a glimpse into the diverse reasons people opt for home education and the various pedagogical methods they employ. Key themes and patterns among the portraits are presented below.

**Portrait 1: The Educational Innovator—Emma.** Emma was chosen for her background in education, innovative approach to home education, and commitment to creating a dynamic and personalized learning environment for her son (aged four) and daughter (aged seven).

***Homeschooling Curriculum and Pedagogies***. Emma’s pedagogical approach is deeply rooted in personalized curricula, experiential activities, and project-based learning. She leverages real-world applications to make learning relevant and engaging. Emma’s home education environment reflects a constant exploration of new teaching methodologies, evolving alongside her young children’s interests and needs (Field notes, 31 November 2022).

Emma and her husband believe that education is a journey of growth with their children, not just memorizing facts. She further elaborated that both have learned a great deal from and alongside their children in the past few years. Subjects and topics that she had never felt comfortable with in the past have now become her new interests and even passions. She stated, “We do a lot of biology and chemistry experiments/projects together… last year, following the children’s interest, we spent five weeks learning about spiders, creatures I was very much afraid of while growing up! We had a lot of fun and learned so much about arachnids and the important roles they play in ecosystems.” (E. Smith, personal communication, 31 November 2022).

***Home Education Artefacts.*** In Emma’s homeschool, one finds an array of art supplies, science experiment kits, and a makeshift outdoor classroom. The dining table doubles as a collaborative workspace where children engage in group projects. The home is adorned with artefacts from their explorations, showcasing the tangible outcomes of their hands-on learning experiences.

The artefacts that Emma shared were very telling of her pedagogical approach and philosophy of life. One particularly notable item she shared during my observation was a 48-piece microscope set, which she and her children used to create a variety of slides for examination (Field notes, 28 November 2022). Emma’s choice of educational tools, such as the microscope set, reflects her commitment to hands-on learning. By providing her children with access to such tools, she encourages exploration, fostering curiosity and a deeper understanding of scientific concepts. This approach enriches their academic knowledge and instils a sense of wonder, aligning with Emma’s holistic philosophy of education.

**Portrait 2: The Faith-Centric Family—David and Sarah.** David and Sarah were selected to represent a religious perspective in home education, illustrating how faith plays a central role in shaping their educational choices for their three young children aged from one to eight.

***Homeschooling Curriculum and Pedagogies.*** The pedagogical approach of David and Sarah is centred on integrating religious teachings into every aspect of their children’s education. They employ a Bible-based curriculum, incorporating scripture studies, moral teachings, and community service. The home education environment reflects their commitment to nurturing a strong spiritual foundation. David expressed, “Our aim is to nurture not only academic knowledge but also a robust moral compass deeply grounded in our faith.” (D. Robson, personal communication, 26 November 2022).

Sarah also shared that they use a Bible-based curriculum in the hope that their three children receive an excellent education and excel not only academically but also spiritually and morally: “We do feel the weight of the responsibility for their future, and we are committed to guiding and supporting them every step of the way.” (S. Robson, personal communication, 26 November 2022)

***Home Education Artefacts.*** The walls of David and Sarah’s home are adorned with religious artwork, and a dedicated prayer space is integrated into their daily routine. The family engages in collaborative Bible study sessions, and community service projects are documented through photographs and journals, serving as reminders of the values they aim to instil. The collection of family photographs that Sarah shared as a unique artefact during the interview is also indicative of their family life and home education. Sarah further clarified:

Now, with home education, everyone in our family is much less stressed. We have been able to spend more time together as a family. We no longer need to rush to catch the school bus or attend events. Instead, we take time to talk, play, and pray. These photos bear witness to our happy life together. They capture the moments of laughter, learning, and love that define our days, serving as a visual reminder of the precious memories we create as we journey through life together.

(S. Robson, personal communication, 26 November 2022)

**Portrait 3: The SEN Advocate—Sophie.** Sophie, a single mother navigating SEN for her six-year-old son Nathan, was chosen to highlight the challenges faced by home-educating parents addressing unique learning requirements.

***Homeschooling Curriculum and Pedagogies.*** Sophie’s home education pedagogy is characterised by flexibility and individualized attention. Sophie’s home is a safe and supportive space where her child’s unique learning needs are addressed with patience and creativity. Sophie emphasised, “Homeschooling enables me to concentrate on my child’s strengths and passions instead of constantly trying to conform to a system that doesn’t comprehend his needs.” (S. Lee, personal communication, 28 November 2022).

When asked about how she managed to teach her son, Sophie shared that she employs adaptive teaching methods, therapeutic interventions, and a collaborative approach with special education professionals. She ensures that she dedicates a minimum of 20 h per week to actively engage in her son’s schooling, providing him with the necessary guidance, support, and assistance to facilitate his learning and academic progress. She further commented:

Frankly, I don’t consider myself an SEN expert at this point. As a business owner and a single mom of a young child with disabilities, I often experience a very high level of stress and anxiety. Life is inevitably hectic. However, since my work schedule is flexible, I get to home educate my son, and I’m continually learning and adapting to meet his educational and developmental needs.

(S. Lee, personal communication, 28 November 2022)

***Home Education Artefacts.*** Sophie’s home is equipped with sensory-friendly spaces, and educational tools tailored to her child’s learning style. Adaptive technology and learning aids are seamlessly integrated into their daily routine.

One of the most prominent features is the indoor brain gym, purposefully built to support Nathan’s sensory needs (Field notes, 29 November 2022). When asked to choose an artefact to represent her home education journey, Sophie picked up a sensory cushion from the brain gym. She further explained that the sensory cushion was not only a symbol of their home education journey but also a tangible reminder of the dedication and effort invested in creating a supportive learning environment tailored to Nathan’s unique needs.

**Portrait 4: The Traveling Classroom—Mark and Lisa.** This couple was chosen for their nomadic lifestyle, integrating travel into their children’s education, and providing insights into the global dimensions of home education. They travel with their three young children (aged five–nine) on a regular basis. As a family, they were able to visit places during less expensive and crowded times, allowing them to travel widely and spend more time together. Mark runs his own business and has a highly flexible work schedule, and this is a great advantage to their home education commitment.

***Homeschooling Curriculum and Pedagogies.*** Mark and Lisa’s home education pedagogy was nomadic and experiential. They leverage the world as their classroom, incorporating travel experiences, cultural immersion, and historical exploration into their children’s curriculum. Their approach prioritised hands-on learning and exposure to diverse perspectives (Field notes, 8 January 2023). The remarks from Mark illustrate their perspectives: “Every place we visit is a chapter in our children’s education. Learning happens not just from books but from experiencing the world.” (M. Martin, personal communication, 12 January 2023).

Lisa further commented that at the beginning of their home education journey, they were not sure if teaching their children in a traveling classroom was the best option, especially when their youngest was just two years old. Therefore, they had a trial period for about a year and were able to judge more accurately how their children would benefit from this education model.

Both Lisa and Mark also explained that in their first two years of home education, they typically took day trips and did not travel abroad at all. Now that their children are more independent and accustomed to the traveling lifestyle, they visit many neighbouring countries often and “have never looked back”. The family works together to plan their itineraries, giving the children the opportunity to select the sights they want to visit, the activities they wish to engage in within various local communities, and even the type of caravan they prefer.

For Lisa and Mark, one of the paramount aims of parenting is to foster a lasting familial bond with one another. “Homeschooling from the outset unexpectedly revealed a profound shift in the familial dynamic. It is really encouraging to see how our children love each other; the older ones are always happy to help the younger ones.” Lisa commented (L. Martin, personal communication, 11 January 2023). Mark further shared that their children became more receptive to parental guidance across all facets of life. “For the first time, there was a sense of truly living life and learning together.” (M. Martin, personal communication, 12 January 2023).

***Home Education Artefacts.*** The home of Mark and Lisa served as a hub for global learning, adorned with souvenirs, maps, and mementos from their travels. The children recorded their experiences through travel journals filled with pictures they drew and photos they took, creating a rich tapestry of the traveling classroom (Field notes, 10 January 2023). The artefact they chose is a handmade world map quilt, intricately stitched with colourful threads representing the destinations they explored together as a family.

**Portrait 5: The Social Inclusion Advocate—Tony.** Tony, representing a home educator of neurodivergent children, was chosen to explore how home education addresses social inclusion concerns and fosters a supportive learning environment. Tony’s two sons aged six and eight at the time of the study, both struggled with school refusal issues. Since Tony’s children are both boys and deeply attached to him, he assumes the primary responsibility for their education. Now, he has the authority to determine their exposure and guide their social growth.

***Homeschooling Curriculum and Pedagogies.*** Tony’s home education pedagogy prioritizes socialisation and individualized support. He actively engages his child in community activities, extracurricular programs, and therapeutic interventions. Tony’s home is a hub for social inclusion, where his child is embraced for their unique qualities, fostering a sense of belonging. Homeschooling has allowed them to cultivate strong bonds with both family and local communities. Community members are often invited to special events and celebrations, reinforcing these connections. Tony expressed:

We desire our children to be part of a community that understands and accepts them. While home-educated students are often stereotyped as isolated, homeschooling allows us to foster connections that might have been more difficult to establish in traditional schooling.

(T. Kelly, personal communication, 31 January 2023)

***Home Education Artefacts.*** Tony’s home is equipped with sensory-friendly spaces and a dedicated area for social activities. Family outings, community events, and collaborative projects are documented through a visual diary, emphasizing the importance of social interaction and inclusion in his child’s educational environment, reflecting a broader transition in his parenting and advocacy roles. The artefact they chose is a vibrant collage capturing moments of joyful interaction and shared experiences as their children participate in a community garden project with other volunteers. This symbolizes the value of community engagement and inclusivity in their educational journey.

The five portraitures presented above highlight the vibrant diversity within the home education community. Each family depicted has a unique story, shaped by individual aspirations, values, and circumstances, illustrating the varied motivations for choosing home education. Through these portraitures, we gain insight into each family’s educational journey, witnessing the joys, challenges, and triumphs within the home.

Despite diverse educational approaches, a common thread of intentionality and dedication runs through all these stories—a commitment to fostering a love for learning, diversity, and curiosity, and to nurturing their children’s growth in value-aligned environments. This underscores that education is not one-size-fits-all, but rather a spectrum of possibilities, with each family charting their course guided by love, passion, and a belief in its transformative power. This is further emphasised in the following section, which offers a brief portrait comparison and includes a cross-portraiture analysis.

### 3.6. Portrait Comparison

#### Daily Routines, and Children’s Participation in Learning Activities

**Outside the Homes.** Since all children from these home-educating families are under nine, their schedules and routines include many age-appropriate activities. For example, some children have never attended nurseries or traditional schools, so their parents ensure they engage with peers of similar ages.

On the other hand, these homeschooling families also have diverse and dynamic daily routines shaped by their unique educational philosophies and lifestyles. Typically, mornings start with structured lessons tailored to each family’s focus. For example, Emma’s family often follows the children’s lead and uses innovative teaching methods, while David and Sarah incorporate Bible-based lessons. Afternoons often involve hands-on learning activities outside the home. For instance, after lunch, Mark and Lisa often take their children on short field trips, while Sophie’s child attends specialized programs for additional support. This mix of structured and experiential learning creates dynamic, personalized educational experiences.

In general, these home-educated children actively participate in various educational activities beyond their domestic settings. For example, Emma’s children are involved in community projects and educational outings, while David and Sarah’s children attend religious and community gatherings. Similarly, Sophie’s child engages in playgroups and specialized therapy sessions, interacting with peers who share similar needs. One parent articulated:

Our children don’t just learn behind shut doors and in their pyjamas, as some may think. Together we explore the world around us, go on exciting field trips and participate in community activities. Learning happens everywhere, not just at home.

(E. Smith, personal communication, 29 November 2022)

**Motivations for Homeschooling.** By analysing the portraits, we identified the various motivations driving parents to choose home-educating. One recurring theme is dissatisfaction with the traditional education system. All parents voiced concerns regarding prevalent issues in schools, such as the one-size-fits-all approach, internet safety, sex education, over-reliance on digital devices and technologies, violence, and limited flexibility. Another prevalent motivation is the desire for personalized learning experiences. Portrait narratives consistently highlight parents seeking to tailor education to their child’s unique learning style, pace, and interests. For example, Tony’s decision to homeschool was driven by the desire to provide a nurturing and tailored educational experience that traditional schools could not offer. This change not only aimed to meet his child’s unique needs but also sought to create a learning atmosphere where his child could thrive both academically and socially. Religious convictions also emerge as an important factor, with one family (i.e., David & Sarah Robson) choosing home education to integrate their faith into their child’s education.

Some parents opt for home education because of the freedom it offers. Sarah articulated that the primary motivation for homeschooling is freedom:

I’ve found that the extended days and excessive hours of traditional schooling weren’t conducive to optimal learning for children. My children set their own goals (with my support at times) and can often finish their work for the day within three hours.

(S. Robson, personal communication, 26 November 2022)

Other parents have discovered that with homeschooling, their children learn at their own pace and develop a natural love for learning. For instance, David noted: “I observed that my children grasped concepts more rapidly and were more eager to engage with the material when they had a say in when subjects were tackled.” (D. Robson, personal communication, 26 November 2022).

**Challenges Faced by Homeschooling Parents.** The challenges faced by home-educating parents are multifaceted. Curriculum development proves to be a common hurdle, with parents navigating the vast educational landscape to create a well-rounded and comprehensive learning experience. Socialisation concerns arise, as parents deliberate on providing opportunities for their children to interact with peers while maintaining the benefits of a controlled, home-based environment. Balancing the roles of parent and teacher is also a common challenge, needing careful management of dynamics at home.

**Visions and Expectations**. In line with the theme of parental autonomy in education, parents shared their belief in the freedom for families to educate their children outside traditional schools. Additionally, they expressed support for flexi-schooling, allowing parents the option to blend school and home learning. They advocate for easily accessible homeschooling resources through local schools, the inclusion of home-educated children in assessments, and the provision of government funding. Below are more insights from parents:

Each state-educated child costs taxpayers about £8000 annually, if I remember correctly. Home-educated students shouldn’t be disadvantaged. Despite paying taxes, we bear the cost of our children’s education independently, often without being able to afford private tuition. I suggest home educators receive a grant matching state education costs.

(T. Kelly, personal communication, 10 January 2023)

### 3.7. Viewing the Findings through the Lens of Life Course Research

After examining the portraits, it became evident that participants’ diverse life experiences not only influenced their home education practices but also shaped distinct life courses. For instance, the trajectories individuals followed, significant life transitions and pivotal turning points were intricately linked to various facets of their home education journeys, including personal passions and concerns, career pathways, and visions for the future. Upon identifying emerging patterns, we recognized that these findings would likely hold significant implications for both policy and practice in the field. Therefore, we conducted an additional analysis through the lens of life course theory [32]. The following sections will present the dominant patterns that emerged from the data.

**Emma’s Life Course.** Emma’s educational journey has been marked by a relentless pursuit of innovative learning experiences. Trained as an educator herself, she navigated the traditional teaching landscape before realizing the limitations it posed on nurturing creativity and individuality.

***Turning points.*** The pivotal moment for Emma was witnessing her eldest child struggling within the confines of a standardized curriculum. This experience ignited a commitment to redefining education for her children. She spoke of the discovery of home education through an American family living in the UK and seeing it as an option for her children being an important turning point in life. For Emma’s family, homeschooling for the first time was life-changing, as it impacted their family’s relationships, lifestyle, careers, finances, and visions for their children’s future, as well as their outlook on life. She chose to home-educate to create a dynamic and adaptive learning environment that emphasizes exploration, critical thinking, and hands-on experiences.

***Career pathways.*** Emma’s career pathway involved transitioning from a traditional teaching role to becoming an advocate for education alternatives and innovative learning. She has taken on roles such as an educational consultant and curriculum developer and even started her own educational initiative.

***Transitions.*** Emma experienced a significant transition when she shifted from a traditional teaching job to home education. This marked a shift not only in her career but also in her role as an educator and a parent, embracing a more dynamic and adaptive approach to learning.

**David and Sarah’s Life Course.** David and Sarah’s life course is intertwined with a steadfast commitment to their faith. Both raised in religious households, they sought to impart the values and teachings of their faith to their children. Their journey involves a shared commitment to community engagement and spiritual growth.

***Turning Points.*** The decision to homeschool was a natural extension of their faith-centric lifestyle. David and Sarah experienced a turning point when they felt that mainstream education lacked the spiritual dimension, they deemed essential for their children’s holistic development. Another crucial turning point occurred when David’s niece developed a school phobia caused by school violence involving knives, bullying and negative peer pressure. This led to public school withdrawal after five years.

It appears that homeschooling became a means to align their educational approach with their deeply held religious values.

***Career Pathways.*** David and Sarah, influenced by their faith, pursued careers in pastoral work and community outreach within their religious community. They now lead a prominent charity organization in the country.

***Transitions.*** The decision to homeschool was a pivotal transition for David and Sarah, moving from being consumers of mainstream education to active participants in their children’s educational journey. This transition reflects a deeper integration of their faith into their daily lives.

**Sophie’s Life Course.** Sophie’s life took an unforeseen turn when she became a single mother to Nathan, who has SEN. With all the challenges that come with raising a child with SEN, it became a significant factor in her marriage breakdown, leading to divorce when Nathan was about two. This experience led Sophie to independently navigate the inadequacies of mainstream education for her child’s unique requirements, profoundly influencing her views on education.

***Turning Points.*** Sophie’s turning point was the realization that her child’s potential was not limited by their SEN but rather by the constraints of traditional schooling. Homeschooling emerged to tailor educational strategies to address her child’s challenges and provide an inclusive learning environment.

***Career Pathways.*** Sophie’s life course has led her to become an advocate for SEN. She pursued a career in special education and therapeutic interventions and even started initiatives focused on improving the educational experiences of children with SEN.

***Transitions.*** Sophie experienced a life-altering transition when she became a single mother to a child with SEN. This marked a shift in her roles and responsibilities, leading her to navigate the challenges of mainstream education and eventually choose home-educating her child.

**Mark and Lisa’s Life Course.** Mark and Lisa share a life course characterized by a love for adventure and exploration. Their journey together involved embracing diverse cultures and experiences and a commitment to providing their children with a global perspective.

***Turning Points.*** The decision to homeschool became apparent when Mark and Lisa observed the limitations of traditional education in fostering a global mindset. The realization that learning extends far beyond the confines of a classroom prompted them to integrate travel into their children’s education. Mark further explained:

One major turning point in our lives was the discovery of home education and the encounter with homeschoolers in our circle of friends. We recognise the benefits of being taught by certified teachers and learning alongside peers, but we also found that the benefits of parent-led education far outweigh those of traditional schooling. For example, we found that many home-educated children really enjoy and love learning. Their curiosity and motivation to explore and inquire are somewhat rare in many of today’s schools. Home education is a high-reward experience.

(M. Martin, personal communication, 12 January 2023)

***Career Pathways.*** Mark and Lisa’s career pathways involved professions related to travel, cultural exchange, and global education. They worked in fields such as travel writing and international relations and previously ran their own educational travel company.

***Transitions.*** The transition to a traveling classroom marked a shift from a more conventional lifestyle to one focused on embracing diverse cultures. This transition reflects a commitment to providing their children with a unique and globally oriented educational experience.

**Tony’s Life Course.** Tony’s life course is shaped by a commitment to advocating for the well-being of his neurodivergent children. His journey involves navigating the complexities of mainstream education and seeking inclusive environments that nurture his children’s social and emotional development.

***Turning Points.*** Tony’s turning point came during the COVID-19 pandemic. As a parent, he felt compelled to try home education and was surprised to discover that it provided him with clarity on the optimal way to educate his children. He recognised the limitations of conventional education in meeting his children’s social inclusion needs. Homeschooling became a means to create a supportive environment where his children could flourish socially while receiving tailored academic support.

***Career Pathways.*** Tony’s career pathway involved roles related to advocacy and support for neurodivergent individuals, such as working with non-profit organizations, educational advocacy, and creating resources for inclusive education in local schools.

***Transitions***. Tony underwent a significant transition in his life when he struggled with his mental health as a teenager. He was doing well academically but did not have any close friends at school. His second major life transition occurred when he became the father of neurodivergent children. This experience allowed him to reflect on his own school experiences and ultimately recognise the limitations of traditional schooling for his children. Choosing home education represented a shift towards a more inclusive and supportive environment.

## 4. Discussions

### 4.1. Summary of Findings

As previously mentioned, the UK has seen a growing interest in home education as an alternative to traditional schooling [4]. Amidst this context, a new conceptual framework was developed combining the portraiture approach [30] and the life course theory [31,32] to study five families with young children in the UK. Each research question was answered adequately, exploring key motivations for choosing home education over traditional schooling, how families structured their educational practices and daily routines, and the challenges, visions, and expectations families experienced in the context of home education.

The study revealed diverse motivations driving parents to choose home education, including concerns about the traditional education system, a preference for personalized learning, and personal lifestyle choices and ideologies which is congruent with a number of findings in the literature [15,16,17,18]. The research data presented suggests that participants had diverse and dynamic daily routines shaped by their unique educational philosophies. In general, parents consistently sought out beneficial situations to foster their children’s social development. Challenges participants faced included curriculum development, financial burdens, and managing dual roles within the home. However, participants viewed these challenges as worthwhile because their main aim of educating their children in a purposeful manner was being met. In line with the theme of parental autonomy in education, parents shared their belief in the freedom for families to educate their children outside traditional schools. They also expressed support for flexi-schooling, which allows parents the option to blend school and home learning. They advocated for easily accessible homeschooling resources through local schools, the inclusion of home-educated children in assessments, and the provision of government funding. These findings are largely consistent with previous research [13,15] and provide further evidence supporting the purpose of the study.

### 4.2. Implications for Educational Policies and Practices

Drawing on the findings, we discuss the implications for educational policies and potential areas of support for home-educating families within the UK context.

The findings of this study have significant implications for educational policies in the UK. Policymakers must recognise the diverse motivations driving an increasing number of parents toward home education and consider avenues to support this alternative educational choice. Increased support, access to public school resources for home-educating families, as well as the importance of recognizing home education as a valuable educational choice should be key considerations in policy development.

***Addressing Stereotypes and Misconceptions*.** By presenting authentic narratives through real-life portraits, this study challenges stereotypes surrounding home education and contributes to a more nuanced understanding of the diverse motivations and experiences of home-educating parents. Dispelling misconceptions about the motivations and capabilities of home-educating parents is crucial for deepening understanding within society and the educational system.

***Future Research Methods and Directions*.** Another important implication of the study stems from the uniqueness of the research methodology. To the best of our knowledge, this is the first study on home education in the UK that utilized the portraiture method. The portraiture approach has provided a rich tapestry of insights. Future studies should continue to utilize real-life cases as vehicles to explore home educators’ motivations, triumphs, and challenges.

Furthermore, analysing the data from a life course perspective has provided a nuanced understanding of the participants’ home education journeys. By considering the impact of past experiences, milestones, and significant life events, we gain insights into the factors that have shaped their educational choices. This approach allows for a comprehensive exploration of the dynamic interplay between personal histories and current home education practices. Understanding the life course perspective enriches our interpretation of the data, revealing patterns and influences that may not be immediately apparent when examining home education in isolation.

Recognizing the diversity of parental motivations and the challenges faced in this educational journey and the need for a comprehensive understanding of home education in the UK, we underscore the importance of continued exploration into the dynamics of home education and its impact on education in the UK.

### 4.3. Limitations

While portraiture offers a rich and detailed account of individual experiences and supports our search for deeper meanings, it is crucial to acknowledge its limitations, including potential subjectivity and the difficulty in extrapolating findings to broader populations [40]. Additionally, our experience of connecting with participants’ voices, the use of portraiture as a means of discovery and analysis and a creative approach to present research findings present its own challenges. For example, navigating ethical considerations surrounding representation and interpretation, and ensuring the credibility and rigour of the qualitative research process was challenging. As a result, the small sample size of the study may not capture the full spectrum of home education experiences, and the findings may not be fully applicable to all home-educating families in the UK.

## 5. Conclusions

As discussed, in the UK, the landscape of education has witnessed a growing interest in home education as an alternative to traditional schooling. This paper aimed to provide an in-depth study of the lives of home-educating families with young children, adopting the portraiture approach to capture the intricate details of their experiences. Despite its limitations, this study serves as a foundational step for extended research on home education. The implications of this study extend beyond the realm of academia. Educational policymakers are urged to consider the diverse needs and motivations of home-educating families when shaping future policies.

## Figures and Tables

**Table 1:** Characteristics of Participants.

Name	Thematic Framework	Number and Age of Children	Location	Length of Home Education Experience
Emma Smith	Educational Innovator	2 (son: aged 4, daughter: aged 7)	England	3.5 years
David & Sarah Robson	Faith-Centric Family	3 (son: aged 5; daughters: aged 6 & 7)	England	5 years
Sophie Lee	SEN Advocate	1 (son: aged 6)	England	4 years
Mark & Lisa Martin	Traveling Classroom	3 (sons: aged 5 & 9; daughter: aged 6)	Scotland	3 years
Tony Kelly	Social Inclusion Advocate	2 (sons: aged 6 & 8)	Scotland	3.5 years

## Data Availability

Data is unavailable to the public due to privacy or ethical restrictions.

## Appendix A. Home Educating Parent Interview Protocol

**Introduction**

Welcome to the interviews, where we will be discussing the lived experiences of home-educating families of young children in the UK. We will meet once a week for about six weeks, each time focusing on different questions listed below. However, based on your unique experiences and settings, additional questions may be asked.

**Background Information**

Name:

Number and Age of Children:

Location:

Length of Home Education Experience:

**Questions about Individual Home Education Experiences**

1. What motivates you to undertake the unique journey of home education?
2. How would you describe your philosophy of education? What experiences (e.g., career, relationships, schooling), events, people, or factors were the major influences on that philosophy?
3. Have you experienced any major changes which may have impacted your decision to home educate? If so, what are they?
4. Tell me about how you teach your child(ren). What pedagogies do you most commonly use?
5. How do you design curriculum for your child(ren)? What influences it? Where does the process begin?
6. How do you structure your educational practices and daily routines? Please briefly describe one or two future scenarios based on these practices and routines.
7. Please choose an artefact or two to represent your home educational philosophy and practices.
8. What are the needs of your children as they relate to their development? What are you trying to achieve with them?
9. What challenges have you faced as a home educator? How do you navigate the challenges inherent in this unconventional approach outside mainstream school settings?
10. In the context of home education, what are your visions and expectations for your child’s education?
11. Please share any thoughts or experiences that may not have been covered by the previous questions/interviews.
